# Odontogenic cutaneous sinus tracts due to infection with nontuberculous mycobacteria: a report of three cases

**DOI:** 10.1186/s12879-020-05015-5

**Published:** 2020-04-21

**Authors:** Ricardo Pérez-Alfonzo, Luisa Elena Poleo Brito, Marialejandra Solano Vergara, Angela Ruiz Damasco, Perla Luiguimar Meneses Rodríguez, Carmen Elena Kannee Quintero, Carolina Carrera Martinez, Ismar Alejandra Rivera-Oliver, Omaira J. Da Mata Jardin, Bárbara A. Rodríguez-Castillo, Jacobus H. de Waard

**Affiliations:** 1 Centro Clínico de Dermatología y Enfermedades Tropicales, Instituto de Biomedicina Dr. Jacinto Convit, Caracas, Venezuela; 2 Servicio de Dermatología, Hospital Universitario de Caracas, Universidad Central de Venezuela, Caracas, Venezuela; 3 Departamento Laboratorio de Tuberculosis. Instituto de Biomedicina “Dr. Jacinto Convit”, Universidad Central de Venezuela, Caracas, Venezuela; 4 One Health Research Group. Facultad de Ciencias de la Salud, Universidad de las Américas, Sede Queri, Quito, Ecuador

**Keywords:** Soft tissue infection, Cutaneous sinus tract, Nontuberculous mycobacteria (NTM), *Mycobacterium fortuitum*, *Mycobacterium abscessus*, *Mycobacterium peregrinum*, Dental unit waterline (DUWL)

## Abstract

**Background:**

Soft tissue or skin infections due to nontuberculous mycobacteria (NTM) have been reported frequently and are mostly associated with trauma or cosmetic interventions like plastic surgery. However, infection with NTM as a result of a dental procedure have rarely been described and the lack of clinical suspicion and a clear clinical manifestation makes diagnosis challenging.

**Case presentation:**

We report on three patients with a facial cutaneous sinus tract of dental origin, due to an infection with respectively *Mycobacterium fortuitum*, *M. abscessus* and *M. peregrinum.* The infection source was the dental unit waterlines (DUWLs), which were colonized with NTM.

**Conclusions:**

Water of the DUWL can pose a health risk. This report emphasizes the need for quality control and certification of water flowing through DUWLs, including the absence of NTM. Our report also shows the need for a rapid recognition of NTM infections and accurate laboratory diagnosis in order to avoid long-term ineffective antibiotic treatment.

## Background

Infections of soft tissue due to nontuberculous mycobacteria (NTM) are initially often missed in the diagnostic microbiology, principally because it takes 3–14 days for mycobacteria colonies to appear on solid culture medium. Many laboratories only monitor cultures for 48–72 h before they are discarded and then reports either that the cultures were negative or there was only a fast growing Gram-positive or Gram-negative bacteria that in reality was a co-infection or contaminant. Moreover, physicians do not often consider mycobacterial infections; do not ask for the isolation of NTM and consequently the definitive diagnosis is delayed for weeks or even months.

NTM infections have been reported frequently and are mostly associated with trauma or plastic surgery and cosmetic procedures. In Venezuela, authors (including ourselves) have reported NTM infection following mesotherapy, acupuncture treatment, breast implants and liposuction [1–5]. As far as we know, infections due to NTM as a result of a dental procedure and presenting as cutaneous dental sinus tract have never been reported in the English medical literature.

A cutaneous dental sinus tract is a channel that leads from the dental focus of an infection to drain onto the face or neck [6]. Although *sinus tracts* of odontogenic origin are usually intraoral, a chronic infection around the apex of a dental root can drain to the skin. These sinus tracts are most commonly found on the chin or in the submandibular area, but they have been found to occur as far away from the oral cavity as the chest [7]. There are numerous case reports of cutaneous dental sinus tracts in the dental and medical literature [8]. These studies in general conclude that it is common to misdiagnose the cause of the facial cutaneous sinus tracts, since specific dental symptoms are usually absent. Therefore, most case studies emphasize that all chronic draining sinus tracts of the face or neck need a thorough dental evaluation. Only a few bacteriological studies of sinus tracts of dental origin have been undertaken, but they found facultative anaerobes, especially *Enterococcus faecalis*, to be the most commonly isolated microorganism [9]. The standard therapy for a dental sinus tract is incising and draining the oral abscess followed by antibiotic treatment. In general, penicillin and amoxicillin with or without clavulanic acid are administered empirically. If the patient has an allergy to penicillin, erythromycin, azithromycin, clarithromycin, or clindamycin can be administered. Here we report three cases of a cutaneous odontogenic sinus tract that were refractory to standard treatments and were diagnosed as an infection due to NTM. We discuss the diagnosis, treatment and source of the infection.

## Case presentation

### Case 1

**Fig. 1:**
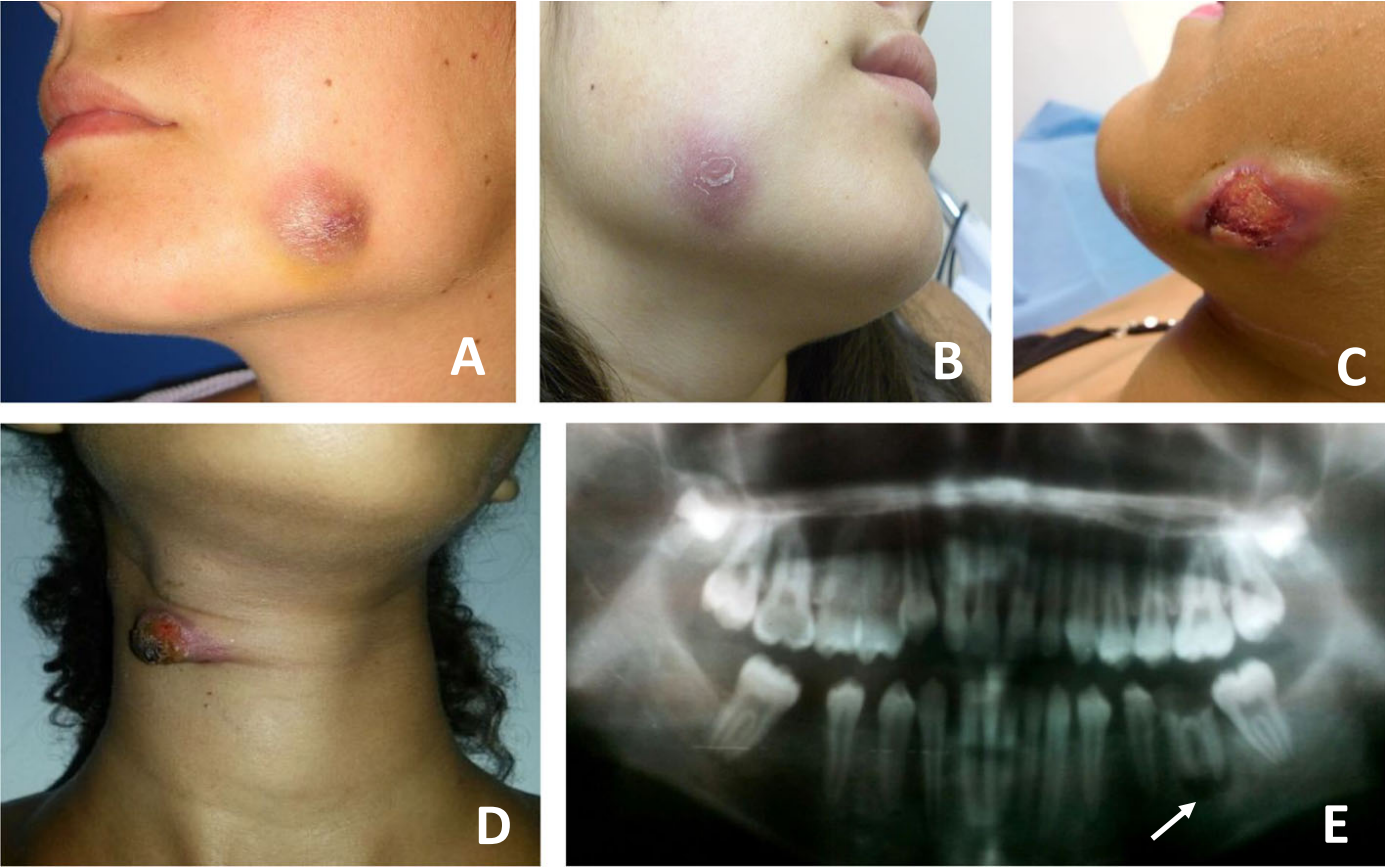
**a-e** The three patients from Caracas, Venezuela visiting our clinic for a diagnosis. **a** and **b** Patient 1 and patient 2 with the diagnosis of a dental sinus tract caused by respectively a *M. fortuitum* and *M. abscessus* infection. **c** and **d** Patient 3 with an infection due to *M. peregrinum*. Shown are the cutaneous facial sinus tract and an affected lymph node draining in the neck. The affected lymph node in the neck was removed with surgery. **e** A panoramic radiography of the patient 3 showing a radiolucent lesion of the periapical area of a mandibular molar (white arrow)

A healthy 21-year-old woman was referred to our dermatology department because she had a tender violaceous plaque with a central draining orifice on the left cheek of more than 2 months of evolution. Six months before, she had her left mandibular third molar extracted and 4 months after this intervention, she developed, a cutaneous erythematous nodule of 3 cm in diameter on her left cheek where the third molar had been extracted (Fig. 1, image A). Dental x-rays showed the presence of a small fistula at the extraction site that connected to the cutaneous lesion. The oral abscess was incised and drained and the patient treated for 10 days with cephalosporin (Cephalexin 500 mg q.i.d.). When she arrived for a check-up, there was a visible improvement and the sinus tract seemed to have resolved itself completely. However, one month later, the patient returned, whereby the lesion was drained and the secretion sent for aerobic and anaerobic bacteriological culture, for the isolation of mycobacteria and fungus. After 48 h of incubation, the bacteriological cultures came back negative. The Ziehl-Neelsen staining of the sample was negative but the culture for mycobacteria on Lowenstein-Jensen medium resulted positive after 5 days of incubation at 37 °C. The isolate was identified as *M. fortuitum* with molecular techniques (PCR-restriction fragment length polymorphism analysis of the *hsp65* gene: the PRA technique).

### Case 2

A healthy 18-year-old woman arrived at the department due to an abscess on her right cheek (Fig. 1, image B). Four months before, she had had her third molars removed and 2 months later, she developed a draining orifice on the right skin. She was empirically prescribed antibiotic treatments with Amoxicillin with clavulanic acid for 7 days and clindamycin for 10 days without any improvement. Magnetic resonance imaging with contrast showed a subcutaneous nodule and a fistula track. Culture for mycobacteria on Lowenstein-Jensen medium, of a serous secretion from the abscess, resulted positive for mycobacteria after 4 days of incubation at 37 °C and the PRA technique identified the isolate as *M. abscessus* type 1.

### Case 3

A 13-year-old girl visited a dental clinic due to a toothache in the left mandibular first molar and was treated with metal instruments (as she self-indicated) and prescribed a 7-day treatment with oxacillin and ibuprofen, presenting clinical improvement. Two months later, the patient visited a clinic again, this time with an erythematous, painful subcutaneous nodule on the left lower jaw (Fig. 1, image C). The panoramic x-ray of the patient showed a radiolucent lesion of the periapical area of the mandibular first molar (Fig. 1, image E) and another antibiotic treatment (Amoxicillin with clavulanic acid for 10 days) was prescribed, which did not resolve the problem. Two months later, due to the persistence of the lesion, the progressive increase in size, and the appearance of a lesion in the neck (Fig. 1, image D), the patient visited our center for evaluation**.** In our department, the lesion was drained and a biopsy was sent for aerobic and anaerobic bacteriological culture for the isolation of mycobacteria and fungus. The bacteriological and fungus cultures came back negative. A culture on Lowenstein-Jensen medium resulted positive for mycobacteria after 6 days of incubation and the isolate was identified as *M. peregrinum* with the PRA technique.

### Laboratory studies

All three patients had normal hematology values, no documented underlying immune deficiency, and were without systemic symptoms like fever when they sought our services. All patients reported a time of symptom onset of between 6 weeks and 3 months after the dental procedure. Susceptibility testing of the isolates was performed according to the Clinical and Laboratory Standards Institute (CLSI) recommendations. The *M. abscessus* strain was also tested for inducible macrolide resistance [10].

### Antibiotic treatment and additional patient care

Following the recommendations of the American Thoracic Society for the management of nontuberculous mycobacterial infection of skin and soft tissue, we treated all three patients using two drugs with in vitro activity against the clinical isolates [11]. Case 1 and 3 were given a daily dose of 500 mg amikacin intramuscular and ciprofloxacin (500 mgr b.i.d) for 3 weeks, followed by ciprofloxacin 500 mg b.i.d during 4 months. For case 2 (*M. abscessus* infection) the quinolone in the two-antibiotic treatment scheme was replaced by clarithromycin 500 mg b.i.d as this strain was resistant to ciprofloxacin and no inducible macrolide resistance was found in a broth micro-dilution assay. In the evaluation of the patients for side effects attributable to aminoglycoside antibiotics, no nephrotoxicity or ototoxicity was recorded. For all patients lesions were weekly drained, or debrided when necessary. Patient 3 had an affected lymph node that drained in the neck (see Fig. 1). This lymph node was surgical removed as antibiotic therapy in general is ineffective for NTM infected lymph nodes [12]. A periodic check-up showed a slow but satisfactory evolution and the patients were declared cured after 5 months of antibiotic treatment. No patients had suffered disease recurrence at a 12-month time follow-up period.

### Looking for the infection source

#### Case 1

We interviewed the maxillofacial surgeon and samples of solutions and water were taken in the dentist’s office to determine the source of infection. Several water samples, taken from the dental unit waterline (DUWL), tested positive for *M. fortuitum*. Upon evaluating the disinfection procedures in the dental office, we registered a regular use of a liquid disinfectant (MicroPlus Action®) for the “sterilization” of critical surgical instruments in between patients and for the disinfection of the DUWL. This product has been registered in our country (Venezuela) as a high-level disinfectant. However, in an earlier publication, we demonstrated that this chemical (5% Polymethylene diurea solution) does not eliminate mycobacteria and for this reason should not be classified as a high-level disinfectant [13]. We deduce that, by improper use of a low-level disinfectant for the decontamination of the DUWL, a biofilm containing mycobacteria had been formed and the patient got infected by contaminated water used during the third molar extraction, which introduced *M. fortuitum* into the wound during irrigation.

#### Case 2

An interview with the dentist was not informative. The DUWL of the dentist chair had never been disinfected and bottled drinking water was used as the source for the waterlines of the chair. Samples of solutions and water were taken in the dentist’s office to determine the source of the infection. The bottled drinking water was free of mycobacterial contamination but the water samples, taken from the DUWL, tested positive for *M. fortuitum* and *M. abscessus*.

#### Case 3

In this case, the infection source is less clear. The patient reported a visit to the dentist, where she was treated with a “metal instrument” as she self-indicated. No follow-up studies were carried out, because the dentist refused to collaborate.

## Discussion and conclusions

We report on three patients with an odontogenic sinus tract due to NTM. The patients’ infections were refractory to a standard treatment with antibiotics, because in general mycobacteria are naturally resistant to these antibiotics, specially to the beta-lactam antibiotics but also to clindamycin or cephalosporins [11]. This underscores the need for the isolation and identification of the causal agent. In addition, treatment of infection with NTM needs prolonged antibiotic administration for periods of up to 5 or 6 months. Our three patients reported a late onset of signs and symptoms of the infection, several months after the odontogenic treatment, which is regarded as typical for a mycobacterial infection that, in addition, usually does not produce systemic symptoms. Manifestations of an infection with NTM generally appear after a delay of some weeks or months and repeated cultures may be needed to isolate the etiologic agent. Consequently, culture for mycobacteria is especially recommended for patients with a late-onset infection without systemic symptoms and when conventional bacterial culture results are negative [14].

Concerning the source of infection, for patients 1 and 2, a water sample from the dental unit waterlines tested positive for respectively *M. fortuitum* and *M. abscessus*, mycobacterial species that were also isolated from our patients. Regarding the dentist’s office visited by patient 1, we believe that, although the dentist had a disinfection program in place for the DUWL, the use of a disinfectant with an incorrect tuberculocidal label claim [13] permitted the formation of a biofilm in the DUWL built up, among other bacteria, of mycobacteria, as the water sourcing the dentist chair was free of mycobacteria. In case 2, the dentist used bottled water and was not aware that water flowing through the DUWL could pose a health risk. He never disinfected the waterlines of the chair and most probably a biofilm had been formed in the DUWL, containing mycobacteria; the bottled water tested negative, but the water in the DUWL yielded mycobacteria. The infection with *M. peregrinum* in case 3 was related to a dental treatment, but no direct evidence linked this case to an infection in the dentist’s office.

The high levels of bacteria in in DUWLs, if no disinfection procedure is in place, is well known. The extent of this problem has been studied in a recent publication from our laboratory. This study was conducted to evaluate water quality in DUWLs in Caracas, Venezuela and Quito, Ecuador, and high levels of NTM were found in most of the units [15]. The identified species included *M. fortuitum*, *M. chelonae*, *M. abscessus*, *M. brisbanense*, and *M. peregrinum.* Furthermore, a publication from Germany found the presence of Mycobacteria in the DUWLs of a dentist’s office [16]. This indicates that high numbers of NTM may be inoculated into oral wounds during dental treatment, creating an infection risk. To prevent microbial *contamination* of DUWL output water, control measures are required. Dental offices should follow current ADA, OSAP, and CDC guidelines [17] and the water quality in the DUWL needs regular microbial control. Also, the products used to prevent the formation of biofilms in DUWLs requires control and certification and need to be high-level disinfectants. It is important to mention that at the moment, there are no guidelines that establish norms for the presence of mycobacteria in water flowing through DUWLs. The minimum water quality requirements for dentists’ facilities do not include the absence of mycobacteria. Concerning the clinical cases described in this article, as far as we know, this is the first report regarding the isolation of NTM from a cutaneous facial sinus tract of dental origin. NTM infections due to a dental procedure are rare and have been reported, as far as we know, only three times and include two outbreaks. An Israeli study in 2005 [18] diagnosed osteomyelitis of the mandible due to *Mycobacterium abscessus* after a root canal treatment. Two outbreaks due to mycobacteria associated with contaminated water from the dental unit water lines have been reported in the USA; one in Atlanta, Georgia and the other in Orange County, California. The first outbreak in 2015 affected 24 children [19, 20]. The outbreak in California, in 2016, affected 72 children [12]. These studies reported infection due to infection with *M. abscessus* after a pulpotomy procedure, the removal of a portion of the pulp of a diseased tooth. In the outbreak in California, also *M. chelonea* was isolated from a patient (personal communication Katheleen O’Donnel). Clinical diagnoses included submandibular and cervical lymphadenitis, mandibular or maxillary osteomyelitis, and pulmonary nodules. No sinus tracts were described in these publications. Our cases are unique in that two cases do not involve the cervical lymph nodes, the usual presentation of Mycobacterium spp. infection after odontological treatment but a sinus tract that has never been described before in the English medical literature.

Concerning the infection source in the USA outbreaks, examination of the water used during the dental procedure showed bacterial contamination far in excess of allowable concentrations, and *M. abscessus* was isolated from all water samples [19, 20]. Water samples set for culture in California also grew other NTM species including *M. chelonae*, and *M. franklinii* (personal communication Katheleen O’Donnel). Also in our study, in Caracas, a strong association was found between DUWL water containing the same NTM species as isolated from the oral infections. A limitation of our study is that no molecular typing of the NTM isolates was performed. This technical demanding molecular technique is not readily available in our country but could provide the definitive evidence that the water of the DUWLs was the infection source.

We conclude that there is a need for quality control and certification of water in DUWLs and that one requirement should be the absence of NTM. Also, a rapid recognition of NTM infections and accurate laboratory diagnosis can avoid a long-term ineffective antibiotic treatment.

## Data Availability

All data is included in the manuscript.

## Abbreviations

NTM: Nontuberculous mycobacteria
DUWL: Dental unit waterline
